# Supplementary material for: Polyketide synthase gene clusters in the ericoid mycorrhizal fungus Oidiodendron maius: genome-wide prediction, expression profiling, and functional characterization of knockout mutants
Source: Front Plant Sci. 2026 Sep 16;17:1924430. doi: 10.3389/fpls.2026.1924430 (PMC13623903; doi:10.3389/fpls.2026.1924430)
Supplement: Supplementary file 1 [file SupplementaryFile1.zip › Supplementary Materials/Supplementary Folder 1.zip!/Oidma1_AssemblyScaffolds_Repeatmasked/knownclusterblast/cluster47/jgi.p_Oidma1_207673_mibig_hits.html]

| MIBiG Protein | Description | MIBiG Cluster | MiBiG Product | % ID | % Coverage | BLAST Score | E-value |
| --- | --- | --- | --- | --- | --- | --- | --- |
| AMR44274.1 | putative\_cupin\_domain\_protein | BGC0001398 | Phomopsins\_biosynthetic\_gene\_cluster | 76 | 96.1111111111 | 272 | 3.6e-73 |
| AAT65712.1 | unknown | BGC0000629 | Aflatrem\_biosynthetic\_gene\_cluster | 66 | 39.4444444444 | 101 | 8.4e-22 |
